# Growth-Promoting Endophytic Fungus (*Stemphylium lycopersici*) Ameliorates Salt Stress Tolerance in Maize by Balancing Ionic and Metabolic Status

**DOI:** 10.3389/fpls.2022.890565

**Published:** 2022-07-11

**Authors:** Raid Ali, Humaira Gul, Mamoona Rauf, Muhammad Arif, Muhammad Hamayun, Husna, Sheza Ayaz Khilji, Aziz Ud-Din, Zahoor Ahmad Sajid, In-Jung Lee

**Affiliations:** ^1^Department of Botany, Abdul Wali Khan University, Mardan, Pakistan; ^2^Department of Biotechnology, Abdul Wali Khan University, Mardan, Pakistan; ^3^Department of Botany, Division of Science and Technology, University of Education Township, Lahore, Pakistan; ^4^Department of Biotechnology and Genetic Engineering, Hazara University, Mansehra, Pakistan; ^5^Institute of Botany, University of the Punjab, Lahore, Pakistan; ^6^Department of Applied Biosciences, Kyungpook National University, Daegu, South Korea

**Keywords:** *Stemphylium lycopersici*, proline, salinity, maize, IAA, endophytic fungus

## Abstract

Climate change is a major cause of the world's food security problems, and soil salinity is a severe hazard for a variety of crops. The exploitation of endophytic fungi that are known to have a positive association with plant roots is preferred for improving plant growth, yield, and overall performance under salt stress. The current study thus rationalized to address how salt stress affected the growth, biochemical properties, antioxidant capacity, endogenous indole-3-acetic acid (IAA), and the ionic status of maize associated with endophytic fungus (*Stemphylium lycopersici*). According to the findings, salt stress reduced chlorophyll a and b, total chlorophyll, total protein, sugars, lipids, and endogenous IAA levels. Enhanced values of chlorophyll a/b ratio, carotenoids, secondary metabolites (phenol, flavonoids, and tannins), antioxidant enzyme activity (catalase, ascorbate peroxidase), proline, and lipid peroxidation were noticed in maize plants under salt stress. Increased ionic content of Na^+^, Cl^−^, Na^+^/K^+^, and Na^+^/Ca^2+^ ratio, as well as decreased Ca^2+^, K^+^, Mg^2+^, N, and P contents, were also found in salt-stressed maize plants. In comparison to the non-saline medium, endophytic association promoted the antioxidant enzyme activities (798.7 U/g protein; catalase activity, 106 U/g protein; ascorbate peroxidase activity), IAA content (3.47 mg/g FW), and phenolics and flavonoids (88 and 1.68 μg/g FW, respectively), and decreased MDA content (0.016 nmol/g FW), Na^+^ ion content (18 mg/g dry weight), Cl^−^ ion (16.6 mg/g dry weight), and Na^+^/K^+^ (0.78) and Na^+^/Ca^2+^ (1.79) ratios, in maize plants under salt stress, whereas Ca^2+^, K^+^, Mg^2+^, N, and P contents were increased in maize plants associated with *S. lycopersici* under salt stress. Current research exposed the role of *S. lycopersici* as an effective natural salt stress reducer and maize growth promoter; hence, it can be used as a biofertilizer to ameliorate salt stress tolerance in crops along with better growth performance in saline regions.

## Introduction

Environmental stresses, including salt stress and arid conditions, intensify these problems and have been demonstrated to reduce agricultural output significantly (Abideen et al., 2021). In the current circumstances, increased food production is required to meet the needs of a growing population, yet climate change, soil contamination, and degradation pose challenges. Inappropriate cultural practices and extensive agriculture applications are promoting soil salinity, leading to a global decline in soil fertility (Hernández, 2019). Plants are affected by salinity stress, which results in morphological, biochemical, physiological, and molecular alterations, as well as a loss in plant growth and productivity. It is also known that long-term saline irrigation causes adverse effects on plant physiology and on the production of plant metabolites that ultimately affect the total biomass production (Hussain et al., 2019). Under salt stress, the uptake of Na^+^ and Cl^−^ ions is increased, resulting in a reduction in the uptake of water and other essential elements (calcium, potassium, magnesium, nitrogen, and phosphorus) as well as resulting in a deficiency of essential ions, disrupted ionic homeostasis, and a reduction in cell division, elongation, root, and leaf growth. Plants use redox balance and sequestration, osmoprotectant and appropriate solute production, enzymatic antioxidants activity, antioxidant substances production, polyamine formation, nitric oxide (NO) production, and hormone regulation to offset the negative effects of salinity (Van Zelm et al., 2020). The most important cereal crop maize (*Zea mays* L.) is ranked third behind wheat and rice, in tropical, sub-tropical, and temperate parts of the world. In comparison to other developing countries throughout the world, Pakistan's average maize output is poor. Nevertheless, important reasons attributed to low productivity include inadequate irrigation caused by water shortage, unbalanced macroelements and microelements, and abiotic stressors, in addition to varieties available to farmers (Shahid et al., 2020). The stressors to which Pakistan's maize crop is subjected are mostly calcareous in origin. Plants that are salt tolerant use a variety of approaches, ranging from morpho-anatomical to physiological and biochemical. Tolerant plants manage turgidity and adjust osmotically by combining severe water dissolvable excellent osmolytes (e.g., glycine betaine, free proline, and low atomic weight sugars) (Ait-El-Mokhtar et al., 2020). Free proline reduces salt-induced oxidative stress in plants, and both reducing and non-reducing sugars help plants maintain turgor under salt or water stress. Among the nutrients, K^+^ plays a key role in the transfer of nitrates to the roots and shoots, increased quick N-digestion, and support for water potential, all of which contribute to plant survival under ecological stresses (Huang et al., 2017). Maize is utilized in a variety of foods and is essential in many facets of daily living. Protein, edible oil, starch, and sugar are all abundant in maize, and it is an important source of biofuel, sugar, cooking oil, animal feed, ethanol, and food all over the world. More recently, putrescine (Gul et al., 2018) and inulin (Gul et al., 2019) have also been found to induce salinity tolerance in maize plants by modulating the physiological, antioxidant, and ionic status. However, the exploitation and bulk production of these compounds are time-consuming, costly, and in most cases, harmful to the environment. In addition, the efficacy of these chemical fertilizers, priming mediators, and growth regulators varies depending on the environmental stress and plant type. Therefore, scientists all across the world are working on low-cost, quick-to-implement strategies to boost agricultural output and sustainability under various environmental stresses. In this context, using beneficial endophytic fungal isolates with growth-promoting abilities to boost agricultural production and sustainability under salt stress provides an option (Ali et al., 2022). Recent research has shown that plants form associations with various microorganisms through their roots and above-ground portions, particularly endophytes, which can benefit plants in both normal and stressful conditions (Yu et al., 2019). Fungal endophytes have been shown to benefit host plants by supplying secondary metabolites and increasing tolerance after boosting the defense system against various abiotic stressors and infections. Different problematic features of microbiota are being addressed nowadays, starting with their identification, to create beneficial associations with plants and increase plant performance, particularly in plant yield and biomass. Symbiotic fungi are thought to be useful for plant adaptation to various stress stimuli, with their physiological effects and molecular mechanisms yet to be discovered (Aziz et al., 2021a,b). Endophytic fungus colonization of host plants is thought to contribute to host plant adaptation to living and non-living stress components. The mechanisms behind positive influence driven by the endophytic fungus *Stemphylium lycopersici* (PW) in maize upon salt stress have not been studied so far. Therefore, the current study was aimed to investigate the potential of *S. lycopersici* (PW) as a biofertilizer for growth promotion as well as stress tolerance amelioration in maize. The current research was focused to explore whether (i) *S. lycopersici* (PW) may induce salt stress tolerance in maize and what could be the possible factors inducing the salt stress tolerance in maize in association with *S. lycopersici* (PW), (ii) the growth-promoting metabolic reshuffling in maize plants upon salt stress, (iii) the modulation of growth-controlling phytohormones, (iv) the antioxidant potential, and (v) ionic rebalancing in the presence of *S. lycopersici* (PW) association. Considering the previous reports and current knowledge, this research was designed to assess the growth-promoting role of *S. lycopersici* on maize plant growth and essential agronomic features, biochemical properties, antioxidant potential, endogenous indole-3-acetic acid (IAA) level, and concentration of various ions while growing under normal and salt stress conditions. Hence, the findings of the current research allowed us to expose the in-depth physiological, biochemical, metabolic, and ionic reshuffling for salt stress tolerance amelioration in maize by *S. lycopersici* (PW) endophytic fungus.

## Materials and Methods

### *Stemphylium lycopersici* (PW) Purification and Spore Suspension Preparation

This experiment used *S. lycopersici* (PW), which was identified in the Plant–Microbe Interaction (PMI) laboratory at Abdul Wali Khan University Mardan (AWKUM). Endophytic fungus PW was isolated from *Chlorophytum comosum* and identified as *S. lycopersici* (data not shown). To purify the fungal strain, it was regularly sub-cultured on potato dextrose agar (PDA) media and then incubated at 25°C for 2 weeks to produce sufficient spores (conidia). Mycelia and spores are cultivated on PDA media with the use of a sterile blade (No. 21) and placed in a sterile conical tube for spore dispersion (50 ml). The tube was then filled with 20 ml of pure water and vortexed for 5 min. The suspension is then filtered with a filter paper (Whatman filter paper, No. 2). With the help of distilled water, the final concentration of spore suspension (5 × 10^7^ spores/ml) was maintained and employed in this experiment.

### Microscopy of the Pure Fungal Colonies

Fungal mycelium was observed under the light microscope (Binocular NSL—CX23 Olympus, Japan) at a low power initially, followed by high magnification, i.e., 40× and 100×. Lactophenol cotton blue reagent was used as a staining agent for investigating the fungal morphological features.

### Experimental Layout

After examining plant growth and biochemistry, an experiment was conducted to examine the performance of *S. lycopersici* (PW) inoculation with the maize plant. After plant inoculation with *S. lycopersici* (PW), the experimental setup was a Completely Randomized Design (CRD). *Zea mays* (Var. Gulibathi) seeds were collected from the Agriculture Research Institute (ARI) in Tarnab, Peshawar. Seeds were sterilized for 60 s with 0.1 % mercuric Cl^−^ before being washed three times with distilled water. The experimental setup consists of a total of 12 plastic pots /treatment (8.5 cm in diameter and 12.5 cm in depth) with a basal outlet (for leaching purposes) and 300 g of sterilized sandy–loam soil. Before filling the pots, soil samples were collected and analyzed for physical and chemical properties (Table 1). Seeds were surface-sterilized with ethanol (70% for 2 min) and perchloric acid (1% for 30 s). After sterilization, final washing was done using double-distilled H_2_O to remove residues of perchloric acid and ethanol. Disinfectant seeds were germinated in glass Petri plates and 4 days after germination, uniformly germinated seedlings were then transferred to the soil pots pre-mixed with the fungal biomass. Liquid spore suspension (5 × 10^7^ spores/ml of *S. lycopersici* (PW) was inoculated (1 ml/seedling and 4 seedlings/pot) for 4 consecutive days. Control was irrigated with an equal volume of irrigation water.

**Table 1:** Physical and chemical properties of soil used in the experiment.

**Soil variables**	**Values**
Texture	Sandy–loam
Sand (%)	73.6
Silt (%)	12.1
Clay (%)	14.1
CEC (dS/cm)	4.1
ECe (dS/m)	1.28
pH	7.1
Organic matter (%)	1.32
Organic carbon (%)	3.98
Carbonates (meq/l)	1.38
Bicarbonates (meq/l)	2.64
Chlorides (meq/l)	1.29

Previously, maize has been known to be moderately sensitive to salt stress, which, however, showed stunted growth up to 200 mM NaCl (Chen et al., 2007). So after 5 days, a 200 mM sodium chloride (NaCl) solution was applied two times a week in control settings with an equal volume of tap water. With five plants per pot, the experimental design was completely randomized with four sets of treatments and was repeated three times.

***Set-I:*** Irrigation of maize seedling performed through tap water (0 mM NaCl).
***Set-II:*** Irrigation of maize seedling performed through 200 mM NaCl.
***Set-III:*** Maize seedlings inoculated with spores of *S. lycopersici* (PW) and irrigation of seedling performed through tap water (0 mM NaCl).
***Set-IV:*** Maize seedlings inoculated with spores of *S. lycopersici* (PW) and irrigation of seedling performed through 200 mM NaCl.

The experiment ended after 35 days, and the maize plants were harvested. The roots and shoots of each harvested plant were carefully separated and rinsed with tap water to remove soil particles. Electrical balance is used to determine the fresh and dry weight of the shoots and roots. The dry weight of the same parts was measured after they had been dried in an oven for 72 h at 60°C.

### Analysis of Primary Metabolites and Photosynthetic Pigments

Primary metabolites (total carbohydrates, proteins, lipids and proline) and photosynthetic pigments (chlorophyll a, chlorophyll b, and carotenoid content) in fresh leaves as described by Ali et al. (2022). Fresh leaves (0.3 g) were ground in 3 ml of 80% acetone before being centrifuged at 11,200 rcf (*g* Force) for 5 min. The supernatant was pooled after the pellet of the plant material was rinsed three times with centrifugation and a final volume of 7 ml was maintained using 80% acetone. Each solution's optical density was measured through UV–Vis spectrophotometer (PerkinElmer Inc., United States) at 663 nm (chlorophyll-a), 645 nm (chlorophyll-b), 480 nm, and 510 nm (carotenoids).

### Quantification of Total Phenols

Total phenolics were estimated as mentioned by Aziz et al. (2021a). With the help of dH_2_O, the extract of the leaf sample was separated in a glass tube, and the volume was increased to 3 ml. The extract was combined with 0.5 ml of Folin Ciocalteau reagent and 2 ml of Na_2_CO_3_. Different concentrations of catechol were used to develop a standard curve. Catechol (CellMark AB, Göteborg, Sweden) concentrations (1–10 mg) were used for developing a standard graph. The absorbance was measured at 650 nm using a UV–Vis spectrophotometer (PerkinElmer Inc., United States).

### Quantification of Total Flavonoids

Total flavonoids were estimated as described by Ali et al. (2021). For extract production, a 5-g leaf sample was macerated in 50 ml of 80% ethanol. After a 1-day incubation period, the extract was centrifuged at 11,200 rcf for 15 min. Then, 250 μl of the extract was combined with 1.25 μl of dH_2_O and 7 μl of a 5% NaNO_2_ solution. After 5 min of incubation, we added 150 μl of 10% AlCl_3_·H_2_O and incubated them for 6 min in the incubator, and 500 μl of 1 M NaOH and 275 μl dH_2_O were added to the solution. Various concentrations (10–100 μg) of Quercetin (TargetMol, Boston, MA, United States) were used for plotting the calibration curve, whereas ethanol (80%) was employed as a reagent blank. The absorbance was measured at 415 nm using a UV–Vis spectrophotometer (PerkinElmer Inc., United States).

### Quantification of Total Tannins

Total tannins in plants were quantified as mentioned by Ali et al. (2022). A 5-g leaf sample was homogenized in 50 ml acetone (70%) and incubated for 6 h in a shaking incubator. From a stock solution of tannic acid (50 g tannin acid diluted in 70% ethanol), serial dilutions were prepared for standard. Then, we mixed 950 μl of dH_2_O with 50 μl o extract and then added 20% Na_2_CO_3_ (2.5 ml) and the reagent (0.5 ml Folin–phenol) to the solution. At 510 nm, the absorbance of the solution was measured against a reagent blank through UV–Vis spectrophotometer (PerkinElmer Inc., United States). Tannic acid (Sigma–Aldrich, St. Louis, MO, United States) was used with various concentrations (10–100 μg) for plotting the calibration curve.

### Lipid Peroxidation Analysis

Heath and Packer (1968) published a lipid peroxidation measurement method in plants using the thiobarbituric acid (TBARS) assay, which was used in this study. In this method, 0.3 g of the plant material was macerated in 2.5 ml of 0.1% trichloroacetic acid and centrifuged at 11,200 rcf for 20 min and 1 ml of the extract was combined with 2.25 ml of thiobarbituric acid and 20% trichloroacetic acid. The solution was heated to 95°C for 30 min and then cooled in an ice bath before being centrifuged for 10 min at 11,200 rcf. The optical density of the solution was measured at 532 and 600 nm through UV–Vis spectrophotometer (PerkinElmer Inc., United States) against the reagent blank.

### Change in Catalase Activity

Chandlee and Scandalios (1984) used a methodology to assess catalase enzyme activity in fresh leaves, and 0.1 ml enzyme extract, 1 ml potassium phosphate buffer (100 mM, pH 7.0), and 0.4 ml H_2_O_2_ (200 mM) were added to the reaction mixture (final volume was 1.5 ml). The absorbance was measured at 240 nm after 30 s intervals (extinction-coefficient = 0.036 mM/cm).

### Change in Ascorbate Peroxidases Activity

Ascorbate peroxidase activity was determined in plants by taking 0.1 ml enzyme extract, 0.2 mM ascorbic acid, 50 mM potassium phosphate buffer, 0.2 mM EDTA, and 20 mM H_2_O_2_, as described by Aziz et al. (2021b). At 290 nm, the reduction in optical density was monitored every 30 s for up to 7 min.

### Quantification of Endogenous IAA Level

Indole-3-acetic acid quantification in plant material was performed using the method mentioned by Aziz et al. (2021b). A 0.5-g leaf sample was ground in 10 ml dH_2_O to make the extract. Then, 2 ml of the Salkowski reagent (was combined with 1 ml of the supernatant), and the solution was incubated in the dark for 30 min before being measured at 540 nm through UV–Vis spectrophotometer (PerkinElmer Inc., United States).

### Analysis of Different Nutrient Content

Various ions (K^+^, Ca^2+^, and Mg^2+^, nitrogen, phosphorus, Na^+^, and Cl^−^ ions) were quantified in 0.3 g of powdered plant samples that were digested using 6.5 ml of acid solution (HNO_3_, H_2_SO_4_, HClO_4_) with a ratio of 5:1:0.5. The solution was heated to the appearance of white fumes. Distilled water was added to the sample for dilution and then filtered with Whatman filter paper, No. 2. The concentrations of ions, in the digested solution, were determined using the ICE 3000 atomic absorption spectrophotometer (Thermo Scientific, United States) as described by Aziz et al. (2021a). Three biological replicates were tested.

### Statistical Analysis

GraphPad Prism 9.0.0 (121) software was used to statistically analyze the data sets that represent the means and standard errors of three independent replicates for each treatment, using the Brown–Forsythe and Welch's test performed to run statistical evaluation through One-Way Analysis of Variance (ANOVA). Data presented as bars with significant differences (*p* ≤ 0.05) marked as asterisks. For results in Table 2, the statistical analysis was performed by using the statistical software package SPSS V. 21.0 (SPSS, Chicago IL, USA) and means separation was carried out by applying the Duncan's Multiple Range Test (DMRT). Significant differences were represented by significant letters and asterisks (*, **, ***) at *p* ≤ 0.05.

**Table 2:** Effect of *S. lycopersici* (PW) on total carbohydrates, total proteins, total lipids, and proline content of maize plants under salt stress.

**Treatment**	**Total carbohydrates**	**Total proteins**	**Total lipids**	**Proline**
	**(mg/g FW)**	**(mg/g FW)**	**(mg/g FW)**	**(μg/g FW)**
Control	78.818 ± 8.983^c^	2.189 ± 0.399^c^	0.717 ± 0.291^b^	0.185 ± 0.107^c^
200 mM NaCl	54.749 ± 0.9^a^	1.417 ± 0.059^a^	0.541 ± 0.003^a^	0.085 ± 0.002^a^
PW	81.850 ± 7.984^d^	2.280 ± 0.245^d^	1.227 ± 0.05^c^	0.145 ± 0.05^b^
200 mM NaCl^+^ PW	59.757 ± 4.951^b^	1.662 ± 0.269^b^	0.570 ± 0.257^a^	0.169 ± 0.003^c^
Probability level	*p* ≤ 0.05	*p* ≤ 0.05	*p* ≤ 0.05	*p* ≤ 0.05

*Means presented with ±SE and followed by different letters are significantly different at p ≤ 0.05. FW, Fresh weight*.

## Results

### Physiochemical Properties of the Soil

Soil mixture was collected from the Mardan district of Khyber Pakhtunkhwa (KPK), Pakistan, for physicochemical analysis. The sand content of the soil mixture ranged from 70 to 74%. The silt content ranged from 10 to 13%. The clay content was 11–15%. Soil pH ranged from 7.1 to 7.9. The electrical conductivity of the soil mixture ranged from 1.2 to 3%. Organic matter was 1.32%, organic carbon 3.98%, carbonates 1.38 meq/L, bicarbonates 2.64 meq/L, and Cl^−^ 1.29 meq/L (Table 1).

### Association of *S. lycopersici* (PW) With Maize Roots and Root Hair Abundance Under Salt Stress

**Figure 1:**
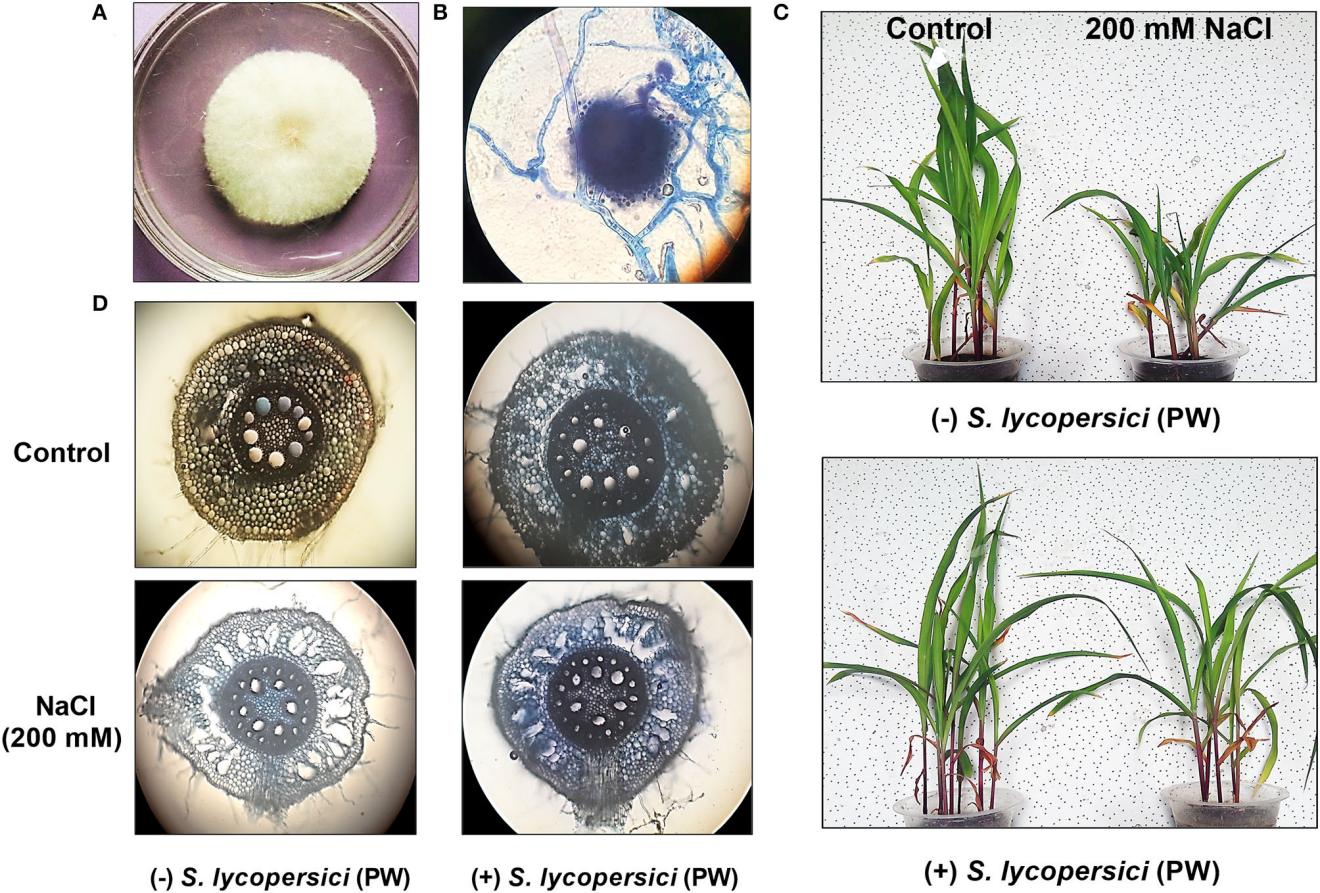
**(A)** Colony appearance of *S. lycopersici* (PW). **(B)** Morphological characteristics of *S. lycopersici* (PW). **(C)** Maize plant growth under normal and salt stress conditions without (upper panel) and with (lower panel) *S. lycopersici* (PW) inoculation. **(D)** Maize root colonization with *S. lycopersici* (PW) and root anatomy under normal and salt stress conditions.

Colonization of *S. lycopersici* (PW) with maize roots is presented in Figure 1D. Root colonization was prominently enhanced in maize grown under saline conditions compared with the control. The occurrence of inoculated endophytic fungal isolate *S. lycopersici* (PW) inside the cortical region as well as the outer zone of maize plant roots was confirmed by microscopic visualization. To further confirm the presence of endophytic fungus *S. lycopersici* (PW) associated with the roots of maize, the endophytic reisolation was performed and its colony morphology was evaluated (Figures 1A,B). The colony phenotype and microscopic morphology further confirmed the association of *S. lycopersici* (PW) with roots of maize plants grown under salt stress.

Moreover, under salt stress, the maize root hairs were found to be less abundantly grown, along with exaggerated and excessive lysogenic aerenchyma formation. On the contrary, a higher abundance of root hairs and moderately formed lysogenic aerenchyma were noticed in plant roots associated with *S. lycopersici* (PW) along with a higher abundance of endophytic fungal hyphae compared with the control (Figure 1D).

### Effect of *S. lycopersici* (PW) on Maize Growth Under Salt Stress

**Figure 2:**
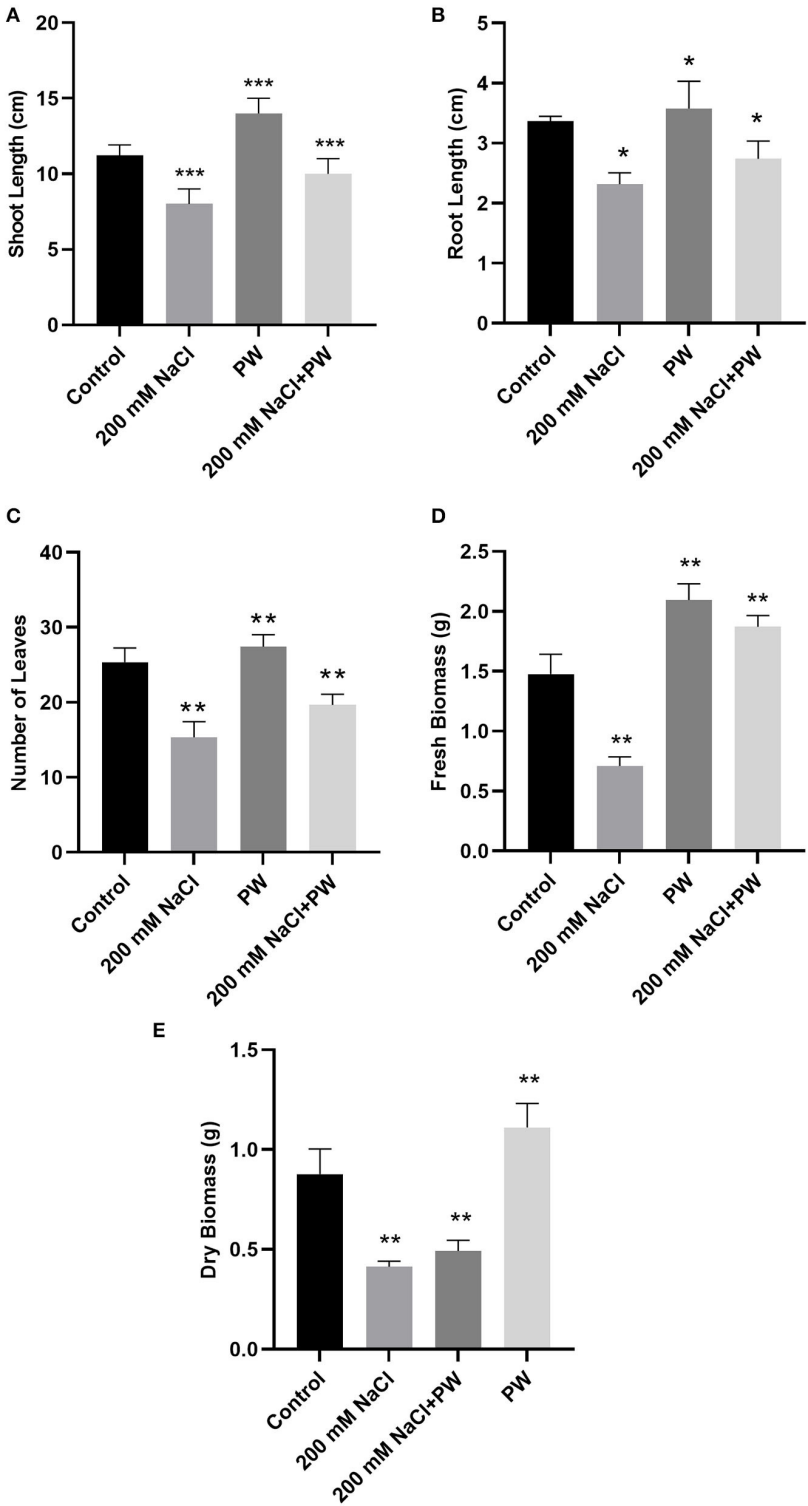
Treatment of maize with *S. lycopersici* (PW) under normal and salt-stressed conditions. **(A)** Shoot length, **(B)** root length, **(C)** number of leaves, **(D)** fresh biomass, and **(E)** dry biomass. Bars indicate mean ± standard error from three replicates at a significant level of *p* ≤ 0.05. Significant differences were represented by asterisks (*, **, ***) at *p* ≤ 0.05.

Maize plants inoculated with *S. lycopersici* (PW) showed a significant (*p* ≤ 0.05) improvement in overall growth response as shown in Figure 1C. The shoot length, root length, and weight were also increased in the maize plants inoculated with *S. lycopersici* (PW) compared to the non-inoculated maize plants under normal as well as salt stress conditions (Figure 2). Assessment of maize plant's growth performance raised under salt stress showed a significant (*p* ≤ *0.05*) reduction in shoot length (−0.26 fold), root length (−0.32 fold), the total number of leaves (−0.4 fold), fresh weight (−0.53 fold), and dry weight (0.54) compared to control plants (Figure 2). Furthermore, *S. lycopersici* (PW) interaction with maize under both saline and normal conditions triggered significant (*p* ≤ *0.05*) positive alterations in morphological traits in comparison to salt-supplemented maize plants. *S. lycopersici* (PW) inoculated plants resulted in an increase in shoot length (0.16 fold), root length (0.13 fold), the number of leaves (0.26 fold), fresh weight (1.65 fold), and dry weight (0.36 fold) of salt-supplemented maize plants compared to non-inoculated maize plants under salt stress.

### Effect of *S. lycopersici* (PW) on Maize Photosynthetic Attributes Under Salt Stress

**Figure 3:**
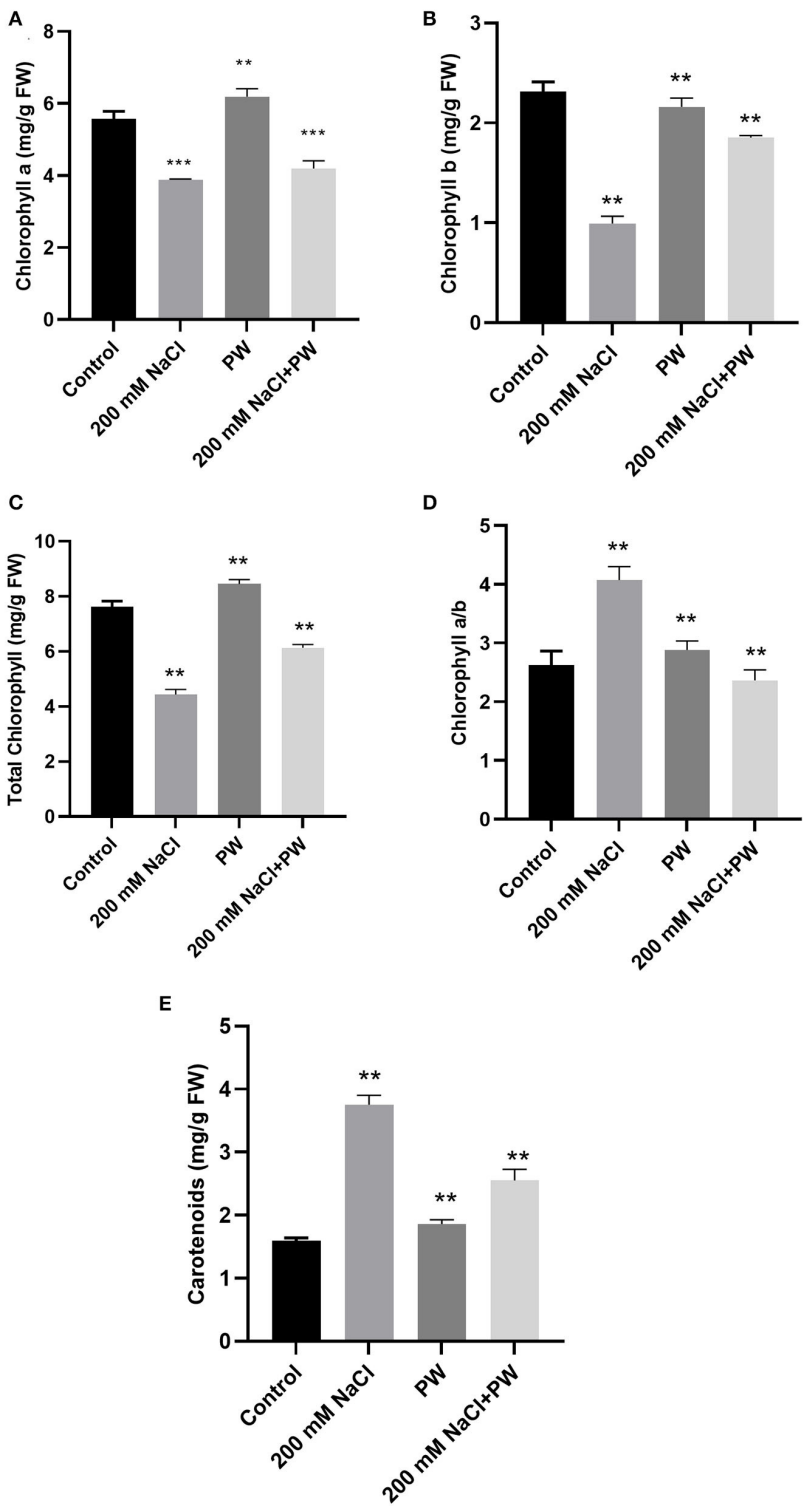
Treatment of maize with *S. lycopersici* (PW) under normal and salt stress conditions. **(A)** Chlorophyll a, **(B)** chlorophyll b, **(C)** total chlorophyll, **(D)** chlorophyll a/b ratio, and **(E)** carotenoids. Bars indicate mean ± standard error from three replicates at a significant level of *p* ≤ 0.05. FW, fresh weight.

The amounts of photosynthetic pigments (chlorophyll a, b and carotenoids) improved significantly (*p* ≤ 0.05) in fungus-inoculated maize plants as compared to the non-associated plants under normal as well as saline conditions (Figure 3). Different photosynthetic pigments of maize plants exposed to salinity stress showed a significant (*p* ≤ 0.05) reduction in chlorophyll a (−0.29 fold), chlorophyll b (−0.56 fold), and total chlorophyll (−0.44 fold) while exhibiting promotion in a/b ratio (0.48 fold) and total carotenoids (1.4 fold) in comparison to control plants (Figure 3).

However, *S. lycopersici* (PW)*-*inoculated plants resulted in a significant (*p* ≤ 0.05) improvement in chlorophyll a (0.1 fold), chlorophyll b (1.9 fold), and total chlorophyll (0.5 fold), and reduction in the value of chlorophyll a/b ratio (−0.4 fold) and total carotenoids (−0.32 fold) of maize plants under salt stress in comparison to non-inoculated plants under salt stress (Figure 3).

### Effect of *S. lycopersici* (PW) on Maize Primary Metabolites Under Salt Stress

Under salt stress conditions (200 mM NaCl), total soluble sugar, total proteins, total lipids, and proline contents of the *S. lycopersici* (PW)-associated maize plants were significant (*p* ≤ 0.05) increased compared to the non-associated plants (Table 2). The biosynthesis of proline and phenolic contents were increased upon salt stress.

Maize plants exposed to salinity stress showed a significant (*p* ≤ 0.05) decrease in total soluble sugars (−0.3 fold), total proteins (−0.36 fold), total lipids (−0.25 fold), and proline (−0.34 fold) compared to control plants (Table 2).

However, *S. lycopersici* (PW) associated plants showed promotion in primary metabolites such as total soluble sugars (0.1 fold), total proteins (1.17 fold), total lipids (0.053 fold), and proline content (0.99 fold) of maize plants grown under salt stress in comparison to non-inoculated plants under salt stress (Table 2).

### Effect of *S. lycopersici* (PW) on Maize Secondary Metabolites Under Salt Stress

**Figure 4:**
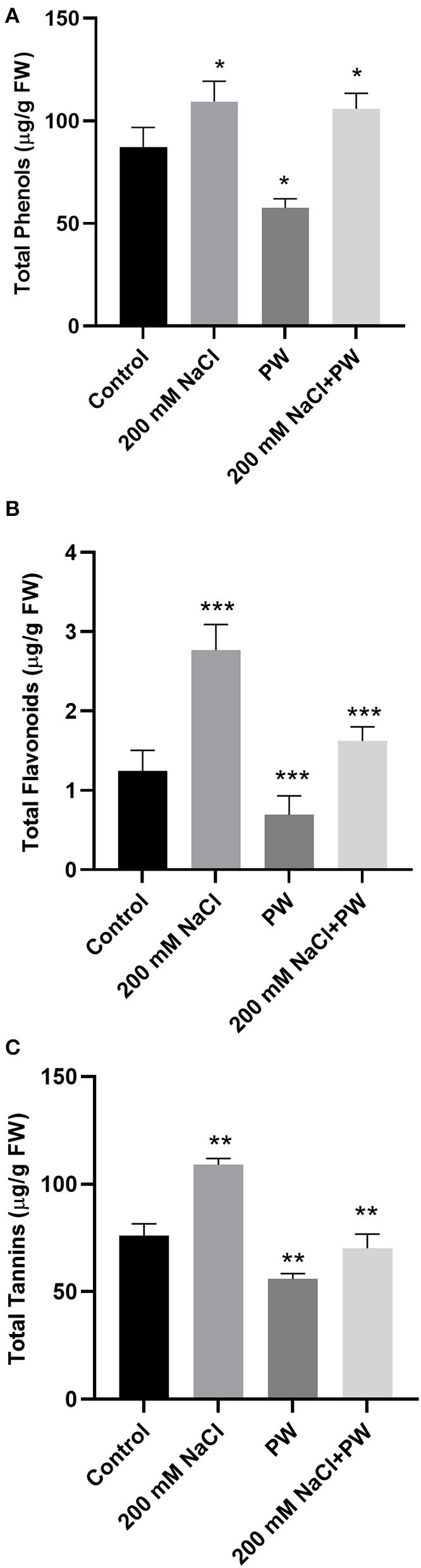
Treatment of maize with *S. lycopersici* (PW) under normal and salt-stressed conditions. **(A)** Total phenols, **(B)** flavonoids, and **(C)** tannins. Bars indicate mean ± standard error from three replicates at a significant level of *p* ≤ 0.05. FW, fresh weight.

Under salt stress conditions (200 mM NaCl), secondary metabolites (total phenols, total flavonoids, and tannins) of the *S. lycopersici* (PW)*-*associated maize plants were significantly (*p* ≤ 0.05) increased compared to the non-associated plants. Maize plants exposed to salinity stress exhibited a significant (*p* < 0.01) increase in total phenols (0.125 fold), total flavonoids (0.97 fold), and total tannins (0.43 fold) compared to control plants (Figure 4).

However, compared with salt-supplemented maize plants, *S. lycopersici* (PW)*-*associated plants exhibited a decrease in the levels of secondary metabolites (−0.1 fold phenols, −0.37 fold flavonoids, and −0.34 fold total tannins) in maize plants under salt stress in comparison to the non-inoculated plants under salt stress (Figure 4).

### Effect of *S. lycopersici* (PW) on MDA Content, Antioxidant Enzymes, and IAA Level of Maize Under Salt Stress

**Figure 5:**
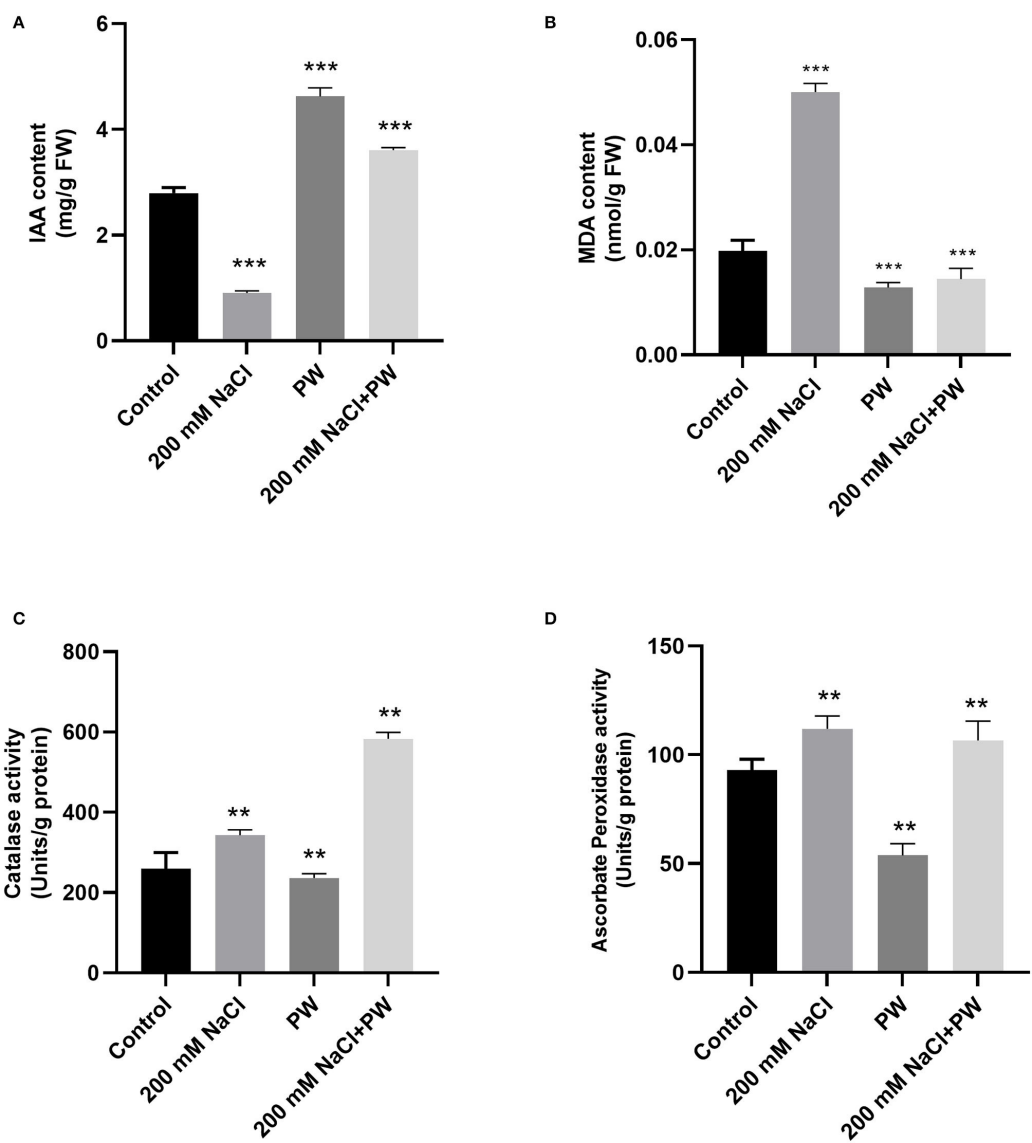
Treatment of maize with *S. lycopersici* (PW) under normal and salt-stressed conditions. **(A)** IAA level, **(B)** MDA content, **(C)** catalase, and **(D)** ascorbate peroxidase. Bars indicate mean ± standard error from three replicates at a significant level of *p* ≤ 0.05. FW, fresh weight.

Adverse effects of high salt stress were observed in maize plants in terms of a significant (*p* ≤ 0.05) increase in MDA content (1.117 fold), catalase (0.33 fold), ascorbate peroxidase activity (0.33 fold), and a reduction in endogenous IAA levels (0.69 fold) compared to control plants (Figure 5).

Whereas *S. lycopersici* (PW)*-*associated plants exhibited a decrease in MDA content (0.6 fold) and an increase in antioxidant enzyme activities (1.35-fold catalase, 0.05-fold ascorbate peroxidase) and IAA content (3.03 fold) of maize plants under salt stress in comparison to non-inoculated plants under salt stress (Figure 5).

### Effect of *S. lycopersici* (PW) on Ionic Contents of Maize Under Salt Stress

**Figure 6:**
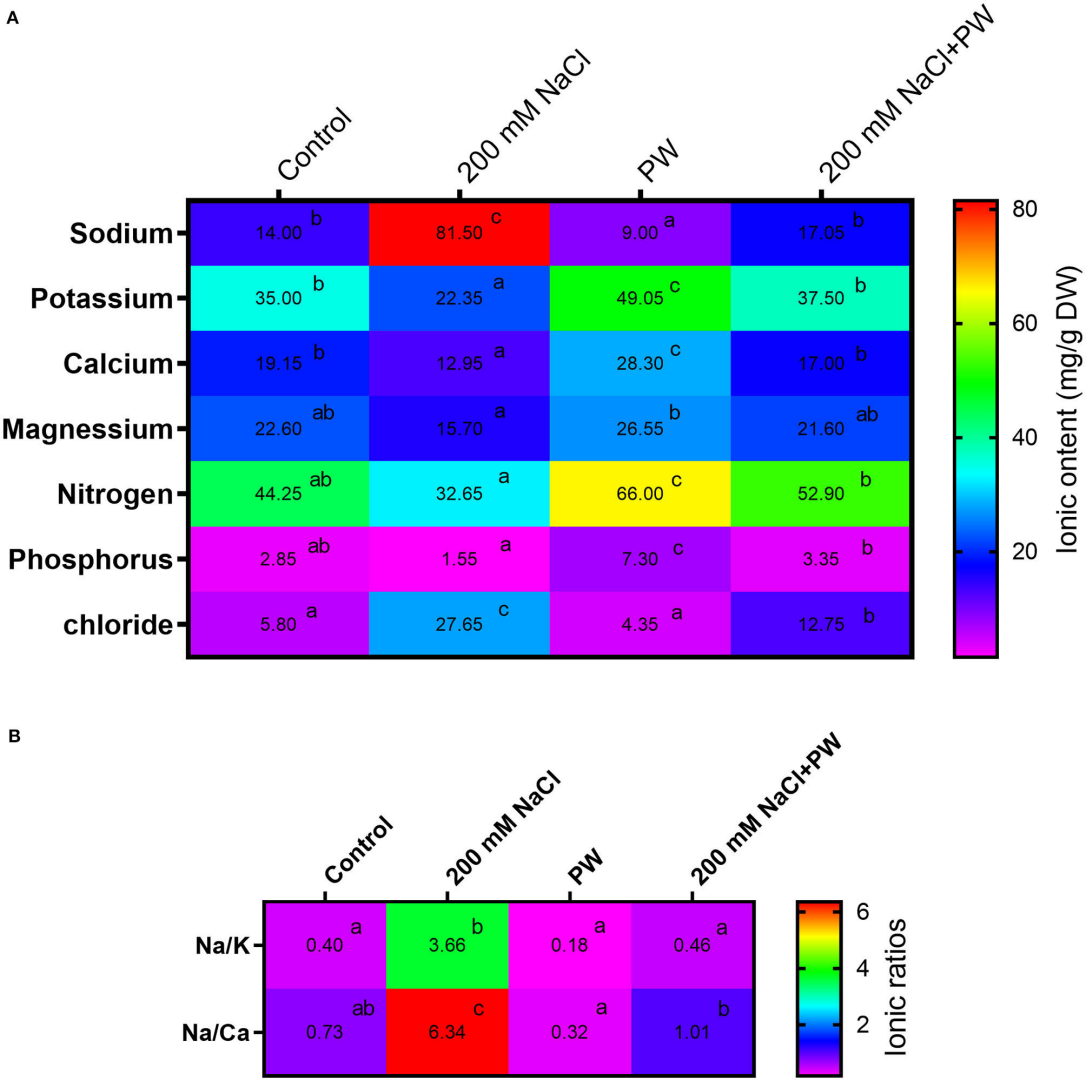
Treatment of maize with *S. lycopersici* (PW) under normal and salt-stressed conditions and their effect on different ions concentrations **(A)** and ionic rations **(B)**. Bars indicate mean ± standard error from three replicates at a significant level of *p* ≤ 0.05. Significant differences are represented by superscript letters at *p* ≤ 0.05.

Current findings revealed the ionic status of maize plants under salinity stress and plants exhibited a significant (*p* ≤ *0.05*) increase in sodium (5.0-fold, 4.5-fold), Na^+/^K^+^ (5.8-fold), and Na^+^/Ca^2+^ (4.8-fold) but showed a significant (*p* ≤ *0.05*) decrease in potassium (−0.35 fold), Ca^2+^ (−0.99 fold), Mg^2+^ (−0.25 fold), phosphorus (−0.84), and nitrogen (−0.5 fold) compared to control plants (Figure 6).

Furthermore, it was observed that the association of *S. lycopersici* (PW) with maize under both saline and normal conditions had significant (*p* ≤ *0.05*) changes in ionic concentrations. *S. lycopersici* (PW) association exhibited reduced ionic content of Na^+^ (−0.8 fold), Cl^−^ (−0.34 fold), Na^+^/ K^+^ ratio (−0.72 fold), and Na^+^/Ca^2+^ ratio (−0.63 fold) and increased ions such as K^+^ (1.5 fold), Ca^2+^ (0.06 fold), Mg^2+^ 0.46 fold), phosphorus (2.47 fold), and nitrogen (0.67 fold) in maize plants under salt stress compared with the non-inoculated plants under salt stress (Figure 6).

## Discussion

At present, endophytic microbe-assisted abiotic stress alleviation is believed to be a valuable strategy for the restoration of sustainable growth of crops under stressful environments. For example, plant growth-promoting rhizobacteria (PGPR) have been used for the amelioration of heavy-metal stress tolerance in plants and may be used effectively for phytoremediation of metal-contaminated sites. For example, the application of *Bacillus megaterium* MCR-8 alleviated nickel stress in *Vinca rosea* (Khan et al., 2017), *Bacillus megaterium* OSR-3 in combination with putrescine ameliorated hydrocarbon stress in *Nicotiana tabacum* (Tariq et al., 2021), *Burkholderia cepacia* CS8 alleviated Cd-stress in *Catharanthus roseus (*Khan et al., 2018), and *Bacillus subtilis* FBL-10 and silicon enhanced nickel stress tolerance in *Solanum melongena* (Shah et al., 2021).

It has also been reported previously that endophytic fungi may also contribute to the enhancement of growth by enhancing biomass, shoot length, and growth-promoting hormones and metabolites under various abiotic stresses (Aziz et al., 2021a,b; Rauf et al., 2021; Ali et al., 2022).

Endophytic fungi have a lot of potential when it comes to mitigating the effects of salt stress, improving plant development, and nutrient uptake. The exploitation of endophytic fungi can give useful and environmentally acceptable solutions for global food security in the long run (Vaishnav et al., 2019). Plant growth-promoting fungal endophyte *Piriformospora indica* alleviates salinity stress in *Medicago truncatula* (Li et al., 2017a), the fungus *Aspergillus aculeatus* enhances salt stress tolerance and metabolite accumulation and improves forage quality in perennial ryegrass (Li et al., 2017b), and *Trichoderma harzianum* mitigates salt stress in cucumber (Zhang et al., 2019).

Salinity generates detrimental environmental and hydrological situations that restrict regular crop production. Roots are the first tissues directly encountering salt stress and acting as its sensor. The root system is liable for water and nutrient intake, which is vital for the development of plant growth and development. The plasticity of roots under salinity stress is the important thing in dealing with stressful situations, as root surfaces first get exposed to environmental stresses. Root morphological plasticity may also encompass stopping the accumulation of salt in roots so that water uptake may also be maintained in saline soils.

Furthermore, plants exposed to progressive salt stress developed morphological changes to mitigate the negative effects of Na^+^. The lysogenic aerenchyma formation due to the replacement of dead cells by air spaces minimizes the uptake of this harmful ion, which is one of the primary anatomical modifications. However, due to increased cell wall thickness, this abundance of such alterations maximizes tissue impermeability and inhibits ionic flow. Thus, plants under extreme salt stress have reduced absorption of water and mineral nutrients, which reduces root and shoot development (Jung and McCouch, 2013).

Second, salt stress in large part regulates the formation and development of root hairs. Salt stress at a decreased concentration (5 g/L NaCl) induced ample root hairs; however, the high salt concentrations (10 and 15 g/L NaCl) progressively decrease the numbers of root hairs, which had been counted. In stressful environments, the root surface had been discovered to be much less in both monocots (e.g., wheat and barley) and in dicots (e.g., Arabidopsis) (Wang et al., 2022).

The current study was meant to identify the efficient role of newly isolated plant growth-promoting endophytic fungus *S. lycopersici* (PW) for its potential to increase maize growth in a saline environment. The extensive colonization and strong association of isolated endophytic fungus *S. lycopersici* (PW) with maize root under normal and salt stress conditions (200 mM NaCl) suggested that the interaction of this endophytic fungus with maize plants has stimulated the host growth through metabolic and ionic rebalancing under salt stress. Current investigations also revealed a higher abundance of root hairs and moderately formed lysogenic aerenchyma in plant roots associated with the *S. lycopersici* (PW) along with the increased level of endophytic colonization, compared with the control. Moderate formation of lysogenic aerenchyma might be the reason for salt stress tolerance that helped to minimize the uptake of toxic Na^+^ ions, while reduced root hairs and a higher abundance of endophytic fungal hyphae supported the nutrient acquisition and translocation from soil to plants. However, a thorough understanding of the role of *S. lycopersici* (PW) in modulating the root system architecture with a special emphasis on root hair traits under salinity stress would be helpful for future maize beading in saline regions of the world.

The enhancement of plant biomass is a key measure of crop performance. Consistently, *S. lycopersici* (PW)*-*associated maize plants under salt stress showed a significant increase in shoot length, root length, number of leaves, and fresh and dry weights compared to control plants, which might be suggestive of improved macronutrient and micronutrient uptakes from the soil by maize roots in the presence of endophytic fungal hyphae that also replicate the nutrient uptake for plants. The inoculation of maize plants with *S. lycopersici* (PW) considerably enhanced maize growth and ameliorated the salt stress tolerance in the present study. The occurrence of inoculated endophytic fungal isolate *S. lycopersici* (PW) inside the cortical region of maize plant roots, and their successful reisolation further strengthens the active role of *S. lycopersici* (PW). As affirmed by previous studies that an endophytic association with roots of plants improved plant performance, the current evidence also indicates that, in comparison to non-inoculated plants, maize plants inoculated with *S. lycopersici* showed an increase in the shoot and root length, total leaf numbers, and fresh and dry biomass under both salt and normal conditions.

Following salt stress, endophyte colonization increased photosynthetic efficiency (Azad and Kaminskyj, 2016). Moreover, changes in the level of the photosynthetic pigment in response to salt stress are considered as a biochemical marker for identifying salt-tolerant behavior in plants. However, carotenoids have been ascribed additional roles in defense mechanisms, such as scavenging singlet oxygen or protecting chlorophylls from the harmful effects of photooxidation processes (Ashraf and Harris, 2013). In accordance, here the maize plants under salt stress also showed a considerable loss in chlorophyll a, chlorophyll b, and total chlorophyll content while the increase in chlorophyll a/b ratio and carotenoids was noticeably observed. Normally, chlorophyll deficiency in plants is caused by the activation of the chlorophyll catabolic pathway as well as a lack of chlorophyll synthesis as a result of photooxidation of these pigments.

Endophytic fungal colonization of roots can also induce significant changes in the relative abundance of major groups of organic solutes, such as changing the composition of carbohydrates, inducing the accumulation of specific osmolytes, such as proline, and facilitating osmotic adjustment. Stress-adapted fungal endophytes can reduce the harmful impacts of salinity by enhancing a variety of other physiological and biochemical plant responses, such as transpiration rate, antioxidant enzyme activity, and osmoprotectant molecule concentrations such as proline and soluble sugars (Moghaddam et al., 2021).

Osmo-protectants (proline) overaccumulation is suggestive of evading the osmotic imbalances in plants under salt stress. Consistently, maize plants showing higher proline levels under salt stress in the presence of endophytic association might be one of the factors contributing to the amelioration of salt stress tolerance. Salt stress-induced growth suppression in cucumber seedlings was shown to entail increases in proline and decreased soluble protein levels, which contribute to osmotic shift, according to Shao et al. (2015). Under salt stress, proline biosynthesis of gene (*KvP5CS1)* expression has been shown to induce in roots, stems, and leaves of *Kosteletzkya virginica* seedlings that played a critical role in salt stress tolerance (Wang et al., 2015).

During the first phase of salt stress, Ma et al. (2021) reported stunted maize growth to appear with a reduced root and shoot system owing to the poor extension of cells. Similarly, the growth of salt-resistant hybrids demonstrated that it was cell wall extensibility, not turgor, that limited cell extension expansion during the initial phase. While in the second phase, plants need more energy to cope with the toxic effect of Na^+^ and Cl^−^ ions with a deficiency of other nutrients under salt stress (Isayenkov and Maathuis, 2019). Because of changes in physiological and metabolic processes in salty environments, as reported by Ullah and Bano (2019), the drop in biomass increased with the increase in salinity. The accumulation of inorganic ions and organic solutes for osmotic readjustment may stimulate dry matter production under the influence of salinity, whereas a decrease in dry matter content at the higher salinity levels may be due to inhibition in the hydrolysis of reserved foods and their translocation to growing shoots (Genc et al., 2019).

Following the previous reports, the present research also showed a decrease in total carbohydrates, proteins, and lipids in maize plants under salt stress. Primary metabolites (carbohydrates, amino acids, and nitrogen) have previously been known to play a vital protective role in salinity acclimated plants, acting as osmolytes (protecting membranes and protein) and ROS scavengers (Sharma et al., 2019). Moreover, in higher plants, the disaccharide sucrose and cleavage products such as glucose and fructose are the central signaling molecules that regulate the expression of genes involved in photosynthesis, respiration, and the synthesis and degradation of starch and sucrose, as well as the growth of sink tissues, carbohydrate translocation, metabolism, and sensing (Slama et al., 2015). Differential expression of genes involved in photosynthesis, respiration, starch/sucrose metabolism, and cell cycle control is triggered by adverse environmental conditions, resulting in optimal carbon and energy use for plant survival under stress. For example, *HEXOKINASE 1 (HXK1)*, the primary glucose sensor, reacts to glucose concentrations under stress and regulates gene expression accordingly. Since invertases are also linked to abiotic stress tolerance, glucose produced by invertase activity activates HXK to control mitochondrial ROS production (Bolouri-Moghaddam et al., 2010). The present investigation also revealed that maize plants associated with *S. lycopersici* exhibited increased carbohydrates, proteins, and lipids levels in both salinity and normal conditions. Zhang et al. (2019) consistently found that endophyte-associated wheat plants growing under salt stress had an increased amount of carbohydrates. Previously, the endophytic association of *A. awamori* increased the level of carbohydrates in mung bean plants under both controlled and salt-stressed conditions. The increased level of soluble sugars in salt stress in *A. awamori*-associated mung bean could be attributed to molecular defense against oxidative damage (Ali et al., 2022). The association of *B. subtilis* combined with AMF has previously affected the activity of enzymes such as nitrate reductase and nitrite reductase under salinity resulting in increased nitrogenase activity. Nitrate and nitrite reductase regulate the conversion of nitrate to ammonia, which results in amino acid synthesis (Iqbal et al., 2015). Salinity also accelerated the lipid peroxidation activity, resulting in increased membrane permeability and ion leakage from cells. Under salinity stress, leaves of *P. indica-*colonized barley plants showed an increase in total lipid proportion as *P. indica* increased the amount of C18:3 fatty acid in the phospholipid fraction extracted from barley leaves in a similar way to salinity (Yu et al., 2020). Such impacts on host plant total lipid composition could be a symbiotic adaptive mechanism mediated by the endophyte to cope with salt stress in unfavorable environmental conditions. Furthermore, under salinity stress, total phenolic and flavonoid content usually operate as secondary antioxidants and free radical scavengers (Khalvandi et al., 2019). Tannins are water-soluble, condensed phenolic compounds with a wide range of sizes found throughout the plant kingdom (Campobenedetto et al., 2020). The present investigation also revealed a considerable increase in total phenols, flavonoids, and tannins in maize plants grown under salt stress. Previously, supplementing both the salinity stress and selenium had a considerable impact on the total phenolic contents of garlic leaves increased dramatically when exposed to salinity stress (90 mM NaCl) (Astaneh et al., 2018). Accumulated evidence indicates that salinity stress increased total phenolic content, flavonoids, tannin, and alkaloid levels, while endophytic antioxidants also help host plants cope with oxidative stress more effectively under salt stress. Consistently, the current study also revealed that inoculating maize plants with *S. lycopersici* improved the levels of plant phenols, flavonoids, and tannins in both salinity and normal environments. In comparison to non-inoculated plants, fungal endophyte inoculation has been reported to increase SOD, APX, and POD activity in plants (Zhang et al., 2019). For example, in the presence of *WSQ* fungal endophyte, the flavonoid content was enhanced in maize plants supplemented with salt, supporting the improvement of the physicochemical characteristics of the host plants under stress (Ali et al., 2022).

Plant cells are damaged by reactive oxygen species (e.g., superoxide radicals, hydroxyl radicals, hydrogen peroxide, and singlet oxygen) produced upon exposure to abiotic stressors such as different metal ions that were employed by molecular oxygen to generate reactive oxygen species in plants (Khan and Bano, 2018). Current observations also confirmed that, in comparison to control plants, maize plants under salt stress showed a considerable increase in membrane lipid peroxidation, catalase, and ascorbate peroxidase activity. Normally, plants exposed to salt stress had more lipid peroxidation than control, and salt stress is known to induce antioxidant enzymes such as catalase (CAT), superoxide dismutase (SOD), peroxidase (POD), and ascorbate peroxidase (APX) activity increased (Umar and Siddiqui, 2018). According to Alen'kina and Nikitina (2020), plants under salt stress showed an increase in ascorbate peroxidase activity. SOD, peroxidase (POD), and catalase (CAT) activity were all higher in endophyte-colonized plants, implying that the coordination of POD and CAT activity, as well as SOD activity, played a key role in the O^2−^ and H_2_O_2_ scavenging process (Ali et al., 2022). The current study also showed that inoculating maize plants with *S. lycopersici* improved the activity of antioxidant enzymes (catalase and ascorbate peroxidase) and the level of lipid peroxidation in both salinity and normal environments. In the plant cells, ascorbate (ascorbic acid) is the most stable and low–molecular weight non-enzymatic antioxidant. Catalase is part of the ROS scavenging enzyme system, and it plays a key role in maintaining redox balance. Plant–fungal interaction increased ascorbate peroxidase activity while lowering lipid peroxidation, electrical conductivity, MDA, and hydrogen peroxide levels but did not affect catalase and peroxidase activities under salinity (Dastogeer, 2018). Increased antioxidant enzyme activities suggest an effective antioxidant response with salt stress protection and symbiosis of the host plant with endophytic strains (Guler et al., 2016).

Phytohormones also act as signaling molecules that can alter metabolic and physiological mechanisms in plants. In the present study, endophyte-associated maize plants under salt stress showed a noticeable increase in endogenous IAA (auxin) levels compared with control and stressed plants. Previously, it is also reported that auxin has a major role in stress adaptation, while high salinity levels cause a reduction in auxin levels in rice plants (Du et al., 2013). In response to salt stress, a decrease in auxin and salicylic acid was observed in plants with a high level of jasmonic acid and abscisic acid. Auxin and abscisic acid play crucial roles in the activity of various enzymes in plants (Kirecci, 2018). Auxin also controls plant processes, including pollen formation, vascular tissue formation, cell cycle, and plant growth and development. Previously, in comparison to non-endophyte-associated plants, cucumber plants exposed to salinity (60 and 120 mM) with their association with *P. formosus* also showed a significant increase in IAA levels (Khan et al., 2012). Endophytic microbes produce hormones that enhance native levels of plant hormones, allowing endophytes to influence plant development and signaling positively (Rauf et al., 2021). Endophytic microbial strains are also known to produce the phytohormones ethylene, IAA, GA, and cytokinins, which affect the growth of host plant cells and tissues directly or indirectly (Aziz et al., 2021a,b).

By interfering with nutrient uptake, translocation, or distribution inside the plant, high salts (Na^+^ and Cl^−^) in the soil hinder nutrient availability. Sodium and chloride ion toxicity can generate ROS, which can destroy cellular functioning, and the accumulation of toxic Na and Cl^−^ is also accompanied by a massive reduction in cytosolic K, with numerous implications for a cell's metabolic activity and viability (Wu et al., 2018; Rubio et al., 2020). Excessive uptake of salt ions should be avoided to preserve a healthy natural balance of nutrients (both macro and micro). The Na^+^/K^+^ ratio is amplified by rising Na^+^ concentrations, which inhibits cytosolic activities, impacting photosynthesis and respiration (Bhat et al., 2020). With NaCl and Na_2_CO_3_ treatments, the K^+^/Na^+^ ratio was increased by 12- and 17-fold, respectively (Lu et al., 2021). In root and leaf tissues, increased Na^+^ competes with K^+^, influencing cellular metabolism. Increased Na^+^/K^+^ ratios in the cytosol disrupt enzyme function, protein synthesis, turgor maintenance, photosynthesis, and stomatal movement (Evelin et al., 2012). Reduced Na^+^ uptake is reported in mycorrhizal plants at high salinity, showing that the AM fungus controls Na^+^ absorption when it becomes harmful to the plant. The increased ionic flux may harm the plant's cell membranes and alter the cell's water potential too. A notable consequence of salt was discovered to be an increase in the Na^+^/K^+^ ratio in the root and shoot tissue of rice plants under salt stress. However, *P. indica* inoculation lowered the Na^+^ content and raised the K^+^ level in rice plants under salt stress (Kord et al., 2019). Furthermore, the foliar Na^+^/K ratio in barley plants was previously discovered to be changed during *P. indica* root colonization under salt stress (Alikhani et al., 2013).

The current study also showed that in comparison to control plants, maize plants treated with 200 mM NaCl showed a considerable increase in sodium, Cl^−^ ions, and Na^+^/K^+^ and Na^+^/Ca ratios, whereas the endophytic inoculation reversed the ionic status by lowering the level of sodium, Cl^−^ ions, and Na^+^/K^+^ and Na^+^/Ca^2+^ ratios in maize plants under salt stress.

The fungal isolates are also known to ameliorate salt tolerance by boosting K^+^ accumulation and lowering Na^+^ concentration. According to Li et al. (2017a), endophytic fungi have favorable effects on ion homeostasis, allowing plants to conserve high K^+^ uptake and minimal Na^+^ accumulation under salt stress. Salt stress is also known to inhibit phosphorus absorption and buildup in the roots and shoots of plants, while the current study consistently revealed that the maize plants under salt stress had a significantly low accumulation of potassium, calcium, magnesium, phosphorus, and nitrogen levels than control plants. An ion flux imbalance is caused by a greater level of salt inside plants. The previous findings showed that, when salinity was introduced, the experimental plants had more Na^+^ and less K^+^ than the infected plants. The amount of Na^+^ in plant leaves increased dramatically, but the number of essential micronutrient ions declined significantly. This may be due to an ionic imbalance, resulting from a disorder in the integrity of the plasma membranes of cells in addition to a converse relationship between Na^+^ and other micronutrient elements (Roshdy et al., 2021). The phosphorus concentration in plant tissues declines as phosphate ions precipitate with Ca^2+^ ions in salt-stressed soil and become unavailable to plants. Nitrogen is absorbed by plants in the form of nitrate (NO_3_) and ammonium (NH_4_) ions *via* specific ammonium (AMTs) and nitrate transporters (NRTs) (Wang et al., 2018). Salt-induced reduction occurs in photosynthetic and respiration rate (Iqbal et al., 2020) and membrane protein disruption, which alters plasma membrane integrity and also affects NO_3_ and NH_4_ absorption (Raddatz et al., 2020). Because NR is a substrate-inducible enzyme, this competition leads to a reduced flow of NO_3_ from the soil to the roots, lowering the enzyme's activity. As a result of the saline stress, the plants' nitrogen content gets reduced (Huang et al., 2018). Magnesium is a component of chlorophyll and is important for the protection of ribosome molecular structure as well as enzyme activity in plant cells. To be salt-tolerant, plants need an adequate amount of magnesium (Javaid et al., 2019). Maize plants after association with *S. lycopersici* demonstrated an improvement in the level of potassium, calcium, magnesium, phosphorus, and nitrogen that might also be contributing to the normal growth of maize plants under salt stress.

## Conclusions

Salinity stress has negative impacts on agronomical and biochemical parameters with an increase in sodium **(**Na^+^) ions in maize plants. The current investigation revealed a significantly reduced negative impact of salinity on *S. lycopersici* (PW)-associated maize plants in terms of enhanced growth, physiochemical traits, growth-promoting and stress-alleviating metabolites, antioxidant potential, IAA level, and rebalancing of nutrient and essential ions. The present research also revealed an abundance of root hairs and moderately produced lysogenic aerenchyma in plant roots associated with *S. lycopersici* (PW) under salt stress, while excessive root colonization by endophytic fungal hyphae assisted nutrient acquisition and transfer from soil to plants. Moderately formed lysogenic aerenchyma plant roots associated with *S. lycopersici* (PW) could be the explanation for salt stress tolerance, which helped plants under salt stress to decrease the intake of hazardous Na^+^ ions. However, a deeper understanding of the role of *S. lycopersici* (PW) in root system architectural management, with a focus on root hair characteristics under salinity stress, will be beneficial for maize farming in salty parts of the world in the future. Hence, the exploitation of *S. lycopersici* (PW) can be recommended as a biofertilizer for increasing maize crop growth, particularly in salt-affected soils.

## Data Availability Statement

The original contributions presented in the study are included in the article/supplementary files, further inquiries can be directed to the corresponding author/s.

## Author Contributions

MR, HG, and MH conceived the idea and designed the experiments. RA, MR, and HG performed the main experiments. MR and MA prepared and reviewed the manuscript. MR, MA, ZAS, and SK analyzed the data. MH and AU-D critically reviewed the manuscript. Husna provided the endophytic fungus used in the current study. I-JL and MH participated in financial and scientific support for the research. All authors contributed to the article and approved the submitted version.

## Funding

This research was supported by the National Research Foundation of Korea (NRF) Grant funded by the Korean Government (MSIT) (No. 2022R1A2C1008993).

## Conflict of Interest

The authors declare that the research was conducted in the absence of any commercial or financial relationships that could be construed as a potential conflict of interest.

## Publisher's Note

All claims expressed in this article are solely those of the authors and do not necessarily represent those of their affiliated organizations, or those of the publisher, the editors and the reviewers. Any product that may be evaluated in this article, or claim that may be made by its manufacturer, is not guaranteed or endorsed by the publisher.
